# Compulsive sexual behavior disorder and paraphilic disorders in a large Brazilian community sample: an online investigation

**DOI:** 10.3389/fpsyt.2026.1807692

**Published:** 2026-10-07

**Authors:** Gustavo Cambraia Do Canto, João Guilherme Almeida Jordani, Nino Cesar Marchi, Daniel Ingrisani Branco, Thiago Henrique Roza, Karine Schwarz, Klaus Michael Beier, Beáta Bőthe, Maria Inês Rodrigues Lobato

**Affiliations:** 1 Programa de Pós-Graduação em Psiquiatria e Ciências do Comportamento, Universidade Federal do Rio Grande do Sul, Porto Alegre, Brazil; 2 Programa Transdisciplinar de Identidade de Gênero, Hospital de Clinicas de Porto Alegre, Porto Alegre, Brazil; 3 Departamento de Psiquiatria, Laboratório de Psiquiatria Digital, Universidade Federal do Parana, Curitiba, Brazil; 4 Institute of Sexology and Sexual Medicine, Charite - Universitatsmedizin Berlin, Berlin, Germany; 5 Department of Psychology, Universite de Montreal, Montreal, QC, Canada; 6 Centre de Recherche Interdisciplinaire sur les Problèmes Conjugaux et les Agressions Sexuelles (CRIPCAS), Quebec City, QC, Canada

**Keywords:** compulsive sexual behavior, paraphilic disorder, sexual health, transcultural adaptation, understudied populations

## Abstract

**Background:**

The International Classification of Diseases 11th Revision (ICD-11) includes Compulsive Sexual Behavior Disorder (CSBD) among impulse control disorders, aiming to standardize diagnostic criteria and improve the understanding of problematic sexual behaviors across cultures. However, few large-scale studies have investigated CSBD and its association with Paraphilic Disorders (PD) in Latin American populations.

**Objective:**

To investigate the occurrence of high-risk CSBD in a large Brazilian community sample, examine its association with paraphilic interests and disorders, and evaluate associated sociodemographic and clinical factors.

**Methods:**

This cross-sectional online study included 5,227 Brazilian adults recruited through convenience sampling between September 2022 and April 2023. Participants completed sociodemographic questionnaires, a Brazilian Portuguese version of the CSBD-19, and a translated inventory assessing paraphilic interests and disorders based on DSM-5-TR criteria. Descriptive statistics, chi-squared tests, linear discriminant analysis, and multivariate backward stepwise logistic regression were performed.

**Results:**

The prevalence of high-risk CSBD (CSBD-19 > 50) was 5.4% (n = 282). The mean CSBD-19 score was 30.4 (SD = 9.7), increasing to 56.4 (SD = 6.1) among individuals classified as high risk. Male sex was strongly associated with high-risk CSBD (OR = 3.32, 95%CI: 2.45–4.50, p < 0.001). Significant associations were also identified for sexual sadism disorder (OR = 2.69, 95%CI: 1.79–4.07), paraphilic disorder involving specific body characteristics (OR = 2.46, 95%CI: 1.49–4.04), zoophilic interest (OR = 2.39, 95%CI: 1.70–3.37), partialism disorder (OR = 2.00, 95%CI: 1.17–3.42), and paraphilia-related distress (OR = 1.94, 95%CI: 1.44–2.61). Linear discriminant analysis demonstrated that the Negative Consequences domain showed the strongest discriminative contribution for high-risk CSBD classification (ω = 1.23).

**Conclusions:**

This study contributes to more inclusive research on compulsive sexual behavior disorder (CSBD) by investigating a large Brazilian sample, including individuals with diverse sexual orientations and gender identities. Using the ICD-11 framework, the estimated prevalence of high-risk CSBD was 5.4%, a rate comparable to those reported for other common mental health conditions. The study also supports the psychometric applicability of the Brazilian Portuguese version of the CSBD-19 for future epidemiological and cross-cultural research in Latin American populations. Further longitudinal and nationally representative studies are needed to clarify causal pathways, psychiatric comorbidities, minority stress influences, and the neurobiological and psychosocial mechanisms underlying the relationship between CSBD and paraphilic disorders.

## Introduction

Compulsive Sexual Behavior Disorder (CSBD) and Paraphilic Disorders (PD) are complex and frequently overlapping concepts within the spectrum of sexual behavior psychopathology. The most recent versions of the World Health Organization (WHO, 2019) and American Psychiatric Association (DSM-5-TR, 2022) diagnostic manuals provide the most comprehensive definitions of both conditions (1, 2). CSBD involves a recurring and persistent patterns of problematic sexual behaviors, also marked by the inability to resist sexual impulses and behaviors, leading to significant distress and personal harm. PD is defined by intense and recurrent sexual fantasies, impulses, or behaviors in response to unusual objects or situations, the focus of which involves others whose age or status renders them unwilling or unable to consent and on which the person has acted or by which he or she is markedly distressed (1).

Recent studies have indicated that individuals diagnosed with CSBD may exhibit higher rates of paraphilic interests or behaviors compared to the general population (3, 4). This raises questions about whether certain paraphilic traits function as risk factors, manifestations, or comorbidities of CSBD (3–5). Furthermore, the compulsive enactment of paraphilic fantasies despite the negative consequences or repeated attempts to stop may reflect a shared neurobiological mechanism with CSBD, rooted in deficits in inhibitory control and reinforcement learning processes (6).

The term ‘paraphilia’ comes from the Greek ‘para’, meaning ‘around’, and ‘philia’, meaning ‘to like’ or ‘to prefer’. The term first appeared in the DSM-II as a subclass of sociopathic personality disorder, as discussed in studies describing the evolution of the terminology used to describe this phenomenon over time (7, 8). However, most individuals with paraphilic interests are not classified as having a mental disorder. It is only considered a paraphilic disorder when the paraphilic interest is recurrent and persistent, is necessary for sexual pleasure, and results in significant distress or impairment of the individual’s psychosocial functioning (1, 2).

The causes of sexual interests, including those that are paraphilic, remain unclear. Various psychological, developmental, environmental, genetic, and organic factors have been explored, yet none of the theories offer a comprehensive explanation of paraphilic behaviors (7, 8). Some risk factors that may contribute to the development of paraphilic interests/disorders have been identified, including being male, young age, specific brain lesions, a prior history of sexual abuse, hypersexuality, and antisocial personality traits (7, 8).

Since 2005, Beier has been applying the “Dunkelfeld Prevention Project,” which aims to actively contribute to the prevention of sexual abuse of minors, offering special treatment options for people who feel sexually attracted to children and are seeking help for behavioral control. Beier et al. developed a self-administered diagnostic inventory to assess the sociodemographic profile and paraphilic fantasies/interests, and disorders of candidates for their preventive therapeutic program (6).

Notably, to establish the diagnosis of CSBD, it is necessary to exclude other possible causes that better explain this condition, such as clinical conditions that can result in increased sexual interest and uncontrolled impulses, for example, manic episodes and the use of certain psychoactive substances. Moral judgment about one’s sexual fantasies and behaviors, which can cause significant individual suffering, even in the absence of a pattern of compulsive sexual behavior, should also be excluded (1, 8).

The ongoing nosological debate surrounding this diagnosis has long been characterized by a lack of consensus, with differences in social, cultural, and generational factors complicating efforts to develop comprehensive operational criteria that take into account the diverse characteristics of different cultural populations (9–13). Various groups of researchers have therefore recently developed protocols to standardize diagnostic criteria by applying them to large populations in different countries (14–17). Considering the new dimensional approach to diagnosing compulsive sexual behaviour disorder (CSBD) set out in the International Classification of Diseases 11th Revision (ICD-11) in 2019, the CSBD-19 scale was developed to evaluate all aspects of this diagnostic entity (14). The scale has been validated across various cultures and population groups, considering the five clinical domains of CSBD: control, salience, relapse, dissatisfaction, and negative consequences (17).

To elucidate the prevalence of CSBD in the population, various studies have been conducted over the past two decades (4, 5, 11). Two recent studies on population-based communities reported CSBD frequencies between 1.8% to 4.8% (18, 19). A recent multicenter study, The International Sex Survey (ISS), which involved over 82,000 individuals from 42 countries, including Brazil with a sample size of 3,579 Brazilian participants, estimated that the prevalence of compulsive sexual behaviors in the Brazilian adult population is 4.2% (17). Historically, there is a lack of data regarding CSBD outside Western, Educated, Industrialized, Rich, and Democratic (WEIRD) countries (11, 20, 21). In part, the absence of an official diagnostic entity for CSBD and valid and standardized tools for its assessment till the development of CSBD-19, as mentioned above, can explain this scenario (14, 21).

Considering this context, this study aims to replicate a previous study (17) on the prevalence of CSBD in Brazil by applying the CSBD-19 Scale to a larger non-probabilistic sample of the Brazilian population. The study also examined positive associations with CSBD, using sociodemographic and clinical variables, in a multivariate logistic regression model, adjusted for specific confounding variables.

This study is limited to identifying positive associations with high risk for CSBD while considering sociodemographic characteristics. We acknowledge, as a limitation, the inability to establish a clinical diagnosis of CSBD using the instruments employed, given that it was not possible to exclude other confounding factors, such as moral incongruence bias and the presence of other comorbid diagnoses. We also recognize that, although the sample size was numerically substantial, the use of a convenience sample precludes the generalization of the findings to the broader Brazilian population. That said, we also investigated whether an association exists between high risk for CSBD and Paraphilic Disorders, considering that, although these are distinct clinical conditions, the treatment of CSBD when co-occurring with a paraphilic disorder may contribute to a lower risk of recurrence of sexual behaviors involving breaches of consent, as well as to a reduction in the psychological distress associated with both conditions. Our hypothesis is that, within the studied population, CSBD is as prevalent as reported in other population-based studies.

## Materials and methods

### Design and participants

The present study employed a cross-sectional, exploratory, and quantitative design, using an online sample of individuals from Brazil. The sample was selected by convenience, via a link hosted on the Google Forms platform, which was widely shared across social networks and academic mailing lists. The link was also shared on social media by a marketing company in all regions of the country, including the less represented Brazilian regions of the North and Northeast. This was done to reduce the occurrence of selection bias. Data collection was conducted according to the best practice principles of Brazil’s General Law on the Protection of Personal Data (Lei Geral de Proteção de Dados Pessoais – LGPD; Law No. 13,709/2018) (22), through a confidentiality agreement and acceptance of consent for participation, in line with the Code of Ethics for Social Research (Law No.14.874, National Research Ethics Commission). The full questionnaire was available for eight months (from September 2022 to April 2023) until the target number of participants was reached. Respondents were estimated to spend approximately 10 minutes completing the form.

The study involved 5,334 individuals over the age of 18 who voluntarily agreed to participate and signed an informed consent form and who were fluent in Brazilian Portuguese. There were no other exclusion criteria.

A total of 107 participants were excluded due to missing values on variables such as gender, net household income, sexual orientation, and other relevant features. Finally, 5,227 adults were included in the data analysis. We conducted a sample size calculation to estimate the prevalence of Compulsive Sexual Behavior Disorder (CSBD) in our target population, assuming a prevalence rate of approximately 5%, according to national studies previously conducted in foreign countries (18, 19, 23, 24).

### Measures

#### Sociodemographic information

The sociodemographic data of the participants were collected and included the following information: age group (18–24, 25–30, 31–40, or 41–80 years old); biological sex (male or female); sexual orientation (heterosexual, homosexual, bisexual, or asexual); gender identity (cisgender male or female or transgender and non binary people); marital status (married, living together, divorced, or single); household monthly income (up to one minimum wage, one to two minimum wages, three to four minimum wages, five to six minimum wages, seven to eight minimum wages, or more than eight minimum wages); education level (up to high school or college education).

#### Compulsive sexual behavior data

Participants were assessed for their risk of CSBD using the CSBD-19, a 19-item screening measure based on the ICD-11 with five diagnostic guidelines/domains: (1) control, (2) salience, (3) relapse, (4) dissatisfaction, and (5) negative consequences (14). A threshold of 50 points has been designated as the cut-off for classifying individuals at high risk of CSBD. At this threshold, the scale demonstrated high sensitivity (98.5%) and specificity (99.1%), with a positive predictive value (PPV) of 76.4%, a negative predictive value (NPV) of 100%, and an accuracy of 99.1% (14). For this study, we translated the scale into Brazilian Portuguese using the Guideline Principles of Good Practice for the Translation and Cultural Adaptation Process for Patient-Reported Outcomes (PRO), published by the International Society for Pharmacoeconomics and Outcomes Research (ISPOR) (25).

Cronbach’s alpha values ranging from 0.90 to 0.94 in community samples indicated high internal consistency for the scale (14).

#### Paraphilic data

The participants were assessed for their paraphilic fantasies and behaviors using an inventory from the *Dunkelfeld Project* based on the diagnostic criteria for paraphilic disorders outlined in the DSM-5-TR (2). With the author’s permission, three psychiatrists from the research team translated and adapted the inventory into Brazilian Portuguese. The full team then reviewed this preliminary text. This version was then sent to a Portuguese-English translator unfamiliar with the original inventory to produce a reverse translation from Brazilian Portuguese to English. This inventory includes diagnostic criteria for paraphilia disorder following DSM-5-TR criteria, including questions about the presence of paraphilic fantasies, temporal criteria (presence of symptoms for at least six months), and criteria related to impairment and distress associated with paraphilias. In data analysis, a Paraphilic Interest was defined as when an individual responded “yes” to the initial screening question of each section that addresses a specific paraphilic interest. Using voyeurism as an example, the question was as follows: ‘Have you ever experienced sexual arousal from the idea of observing other individuals in intimate situations?’ A Paraphilic Disorder was defined as the presence of the relevant paraphilic interest, lasting for more than six months, and accompanied by a positive response to items assessing impairment or distress related to that fantasy/interest (DSM-5-TR, 2022).

### Statistical analysis

A multivariate logistic regression was assigned to investigate the association between clinical-sociodemographic variables and the high risk of CSBD (i.e., CSBD-19 > 50). The standardized coefficients (β), the odds ratios (OR), and their respective 95% confidence intervals (CI), standard errors (SE), and *p*-values were measured to investigate those associations. We operated the backward stepwise regression approach to access statistically significant results, which initiates with a full model and iteratively selects independent variables to use in the final model, withdrawing features that do not satisfy the criteria of *p* < 0.05. To mitigate bias, we ensured the inclusion of confounding variables (age, marital status, biological sex, sexual orientation, and gender identity), thereby allowing for the adjustment of the predictors’ influences concerning these confounders (26).

The descriptive statistics tests were performed employing means (x̄) and standard deviations (σ) for continuous variables and frequency (n) with percentage (%) to depict categorical variables. The bivariate association between the high risk of CSBD and clinical-sociodemographic variables was investigated using the chi-squared test (χ^2^) and the phi coefficient or mean square contingency coefficient (r_φ_) to assess the strength and direction of the relationship between variables, with *p*-values below 0.05 considered statistically significant.

We conducted a linear discriminant function analysis (LDA) to estimate the discriminant function coefficients for each item of the CSBD-19, employing the high risk of CSBD as the criterion variable. This approach entails computing the discriminant function, a linear composite, and calculating weights from a group of multiple predictors, projecting the data onto a lower-dimensional space to maximize the separation of criterion groups. Using these coefficient values, we can infer the relative contribution of each predictor variable (items and domains) to group separation (27).

Data analysis was performed using R (version 4.1.3) and RStudio (version 2023.12.1). The generalized linear models were fitted within the glm R function from *stats* (version 4.1.3) package. The following additional R packages were used: *MASS* (version 7.3-65), *psych* (version 2.4.12), and *tidyverse* (version 2.0.0) (28–31).

### Ethics

The study received approval from the Human Research Ethics Committee at the Hospital de Clínicas de Porto Alegre. It is registered on the AGH platform (project number: 2021-0488) and identified on the Plataforma Brasil (approval number: 52810521.0.0000.5327). The researchers confirm their awareness of and compliance with the General Data Protection Law (Law No. 13,709, dated August 14, 2018) regarding the processing of personal and sensitive personal data used in this research project. The study was conducted in conformity with the Helsinki Declaration.

This research was conducted with funding from public research agencies, including by the state of Rio Grande do Sul Research Support Foundation (FAPERGS) (grant numbers FAPERGS/INCT 17/2551-0000519-8 and Edital 07/2021), by the National Council for Scientific and Technological Development (CNPQ) (grant number 301326/2017-7), by the Coordination for the Improvement of Higher Education Personnel — Brazil (CAPES) — Finance Code 001, Hospital de Clínicas de Porto Alegre (HCPA) / FIPE, and the Postgraduate Program in Psychiatry and Behavioral Sciences of Federal University of Rio Grande do Sul (UFRGS).

## Results

### Sample characteristics and descriptive results

Among the 5,227 participants, women comprised 58.5% of the group, and 76.6% had some college education (**Table 1**). The prevalence of high-risk Compulsive Sexual Behavior Disorder was 5.4% (n = 282), and the mean CSBD-19 score was 30.4 (σ = 9.7) (**Table 2**). The high risk of CSBD was more common in men, with a relative prevalence of 68.1%, in the 31–40 age group (37.9%), heterosexuals (41.5%), and divorced or single individuals (54.6%). The relative prevalence was also substantial among individuals with paraphilic interests (97.9%) and those without paraphilic disorder (53.5%) (**Table 1**). In the high-risk population, the mean score of the questionnaire was 56.4 (σ: 6.1) (**Table 2**). Additionally, the independent presence of a paraphilic disorder (r_φ_ = 0.22; *p* < 0.001) or paraphilic sexual interest (r_φ_ = 0.04; *p* < 0.05) was associated with a high risk of CSBD (**Table 1**).

**Table 1:** Selected clinical and socio-demographic characteristics of the study sample.

Variables	Positive CSBD-19		r_φ_	*p*-value †
	No (n=4,945)	Yes (n=282)		
**Sex †**				**<0.001**
Male	1,975 (39.9)	192 (68.1)	0.13	
Female	2,970 (60.1)	90 (31.9)	-0.13	
Age Interval				
18–24 YO	1,031 (20.8)	61 (21.6)	–	0.81
25–30 YO	1,022 (20.7)	62 (22)	–	0.65
31–40 YO	1,581 (32)	107 (37.9)	0.03	**<0.05**
41 – 80YO	1,311 (26.5)	52 (18.5)	-0.04	**<0.05**
Education Level				
Up to high-school	1,013 (20.5)	66 (23.4)	–	0.75
Some college education	3,786 (76.6)	216 (76.6)	–	- 0.98
Gender Identity				
Cisgender man	1,938 (39.2)	194 (68.8)	0.14	**<0.001**
Cisgender woman	2,864 (57.9)	80 (28.4)	-0.13	**<0.001**
Others	143 (2.9)	8 (2.8)	–	0.96
Sexual orientation †				
Heterosexual	2,722 (55)	117 (41.5)	-0.06	**<0.001**
Homosexual	694 (14)	57 (20.2)	0.04	**<0.05**
Bisexual	1,341 (27.1)	96 (34)	0.04	**<0.05**
Asexual	188 (3.8)	2 (4.3)	–	0.82
Net household income †				
Up to 1 minimum wage	224 (4.5)	10 (3.5)	–	0.53
Between 1 and 2	741 (15)	60 (21.3)	0.04	**<0.05**
Between 3 and 4	1,420 (28.7)	80 (28.4)	–	0.95
Between 5 and 6	929 (18.8)	41 (14.5)	–	0.09
Between 7 and 8	492 (9.9)	32 (11.3)	–	0.51
More than 8	1,139 (23)	59 (20.9)	–	0.45
Marital status †				
Married/cohabiting	2,354 (47.6)	128 (45.4)	–	
Divorced/single	2,591 (52.4)	154 (54.6)	–	
Paraphilia (sexual interest) †				<0.05
Yes	4,640 (93.8)	276 (97.9)	0.04	
No	305 (6.2)	6 (2.1)	-0.04	
Paraphilia (disorder) †				<0.001
Yes	599 (12.1)	131 (46.5)	0.22	
No	4346 (87.9)	151 (53.5)	-0.22	
Paraphilia openness †				0.11
Yes	3,378 (68.3)	206 (73)	–	
No	1,567 (31.7)	76 (27)	–	
Who knows about paraphilic interest/fantasy †				
Acquaintances and friends	2,336 (47.2)	146 (51.8)	–	0.16
Relatives	370 (7.5)	18 (6.4)	–	0.57
Therapist, physician or teacher	258 (5.2)	37 (13.1)	0.08	**<0.001**
Paraphilia discomfort †				<0.001
Yes	683 (13.8)	113 (40.1)	0.17	
No	4,262 (86.2)	169 (59.9)	-0.17	

r_φ_ = phi coefficient; † = chi-squared test; - = no statistical significance. Bold numbers indicate *p*-values considered statistically significant.

**Table 2:** CSBD-19 items and discriminant function coefficients.

Items	ω n=5,227	Total † n=5,227	Negative CSBD † n=4,945	Positive CSBD † n=282
**Control**	0.80	1.4 (0.4)	1.3 (0.7)	3 (1)
Even though my sexual behavior was irresponsible or reckless, I found it difficult to stop.	0.21	1.4 (0.8)	1.3 (0.7)	2.7 (1.2)
I could not control my sexual cravings and desires.	0.27	1.5 (0.8)	1.4 (0.7)	3.1 (0.9)
My sexual desires controlled me.	0.38	1.4 (0.8)	1.3 (0.6)	3.1 (0.8)
**Salience**	0.15	1.7 (0.5)	1.6 (0.8)	2.9 (1)
Sex has been the most important thing in my life.	0.02	1.8 (0.9)	1.7 (0.8)	2.7 (0.9)
I would rather have had sex than to have done anything else.	0.01	1.6 (0.9)	1.6 (0.8)	2.8 (1.1)
When I could have sex, everything else became irrelevant.	0.06	1.7 (0.9)	1.6 (0.9)	3.3 (0.8)
**Relapse**	0.31	1.5 (0.4)	1.4 (0.8)	2.9 (1)
I was able to resist my sexual urges for only a little while before I surrendered to them.	-0.02	1.7 (0.9)	1.6 (0.9)	3 (1)
Trying to reduce the amount of sex I had almost never worked.	0.03	1.5 (0.9)	1.4 (0.8)	2.9 (1)
I was not successful in reducing the amount of sex I had.	0.32	1.4 (0.8)	1.3 (0.7)	2.9 (1)
**Dissatisfaction**	0.07	2.2 (0.6)	2.1 (1.1)	3.3 (0.9)
I had sex even when I did not enjoy it anymore.	0.02	2.5 (1.2)	2.4 (1.2)	3.3 (0.9)
Although sex was not as satisfying for me as before, I engaged in it.	0.005	2 (1.1)	2 (1)	3.2 (0.9)
Although my sex life was not as satisfying as it had been before, I had sex.	0.07	2 (1.1)	1.9 (1)	3.2 (0.8)
**Negative Consequences**	1.23	1.4 (0.5)	1.3 (0.7)	2.9 (1.1)
I did not accomplish important tasks because of my sexual behavior.	0.12	1.5 (0.8)	1.4 (0.7)	3.2 (0.9)
My sexual urges and impulses changed me in a negative way.	0.20	1.3 (0.7)	1.3 (0.6)	2.6 (1)
My sexual activities interfered with my work and/or education.	0.30	1.4 (0.8)	1.3 (0.7)	3.1 (0.9)
My sexual behaviors had negative impact on my relationships with others.	0.24	1.4 (0.8)	1.3 (0.7)	3 (1)
I have been upset because of my sexual behaviors.	0.05	1.5 (0.8)	1.4 (0.8)	2.7 (1)
My sexual activities interfered with my ability to experience healthy sex.	0.05	1.6 (0.9)	1.5 (0.9)	3 (1.1)
I often found myself in an embarrassing situation because of my sexual behavior.	0.19	1.3 (0.7)	1.2 (0.6)	2.4 (1.1)
**CSBD-19 Total Score**	0.13	30.4 (9.7)	28.9 (7.6)	56.4 (6.1)

ω = discriminant function coefficients; † = mean (x̄) and standard deviation (σ). Bold letters indicate the five diagnostic guidelines/domains of the scale.

### Linear discriminant function analysis

**Table 2** presents the coefficients of linear discriminants of the scale. The LDA revealed that the domain of Negative Consequences had the highest contribution, with a coefficient of 1.23. This finding suggests it plays a significant role in distinguishing high-risk CSBD, followed by Control (0.8), Relapse (0.31), Salience (0.15), and Dissatisfaction (0.07), even though the domain of Dissatisfaction had the highest mean (2.2) and Negative Consequences the lowest (1.4). The single item most associated with a greater likelihood of classification into one of the target classes was “My sexual desires controlled me” (0.38). In contrast, the item with the least absolute weight was: “Although sex was not as satisfying for me as before, I engaged in it” with a coefficient of 0.005. These findings indicate that the psychometric properties identified in the original validation study of this scale were preserved following its translation and cultural adaptation when applied to the target population.

### Multi-predictor analysis

The backward stepwise multi-predictor logistic regression model suggested several sociodemographic and clinical variables as potencial significant risk factors associated with the high risk of CSBD (**Table 3**). Specifically, male biological sex (OR = 3.32, 95%CI: 2.45–4.50), sexual sadism as a disorder (OR = 2.69, 95%CI: 1.79–4.07), paraphilic disorder in specific characteristics of the body (OR = 2.46, 95%CI: 1.49–4.04), zoophilic interest (OR = 2.39, 95%CI: 1.70–3.37), partialism as a disorder (OR = 2.00, 95%CI: 1.17–3.42), discomfort associated with a paraphilia (OR = 1.94, 95%CI: 1.44–2.61), voyeuristic disorder (OR = 1.88, 95%CI: 1.21–2.92), net household income between 1 and 2 minimum wages (OR = 1.74, 95%CI: 1.26–2.40), exhibitionistic interest (OR = 1.72, 95%CI: 1.18–2.52), and sexual interest in diaperism/eproctophilia/masks/scatophilia/urolagnia (OR = 1.39, 95%CI: 1.02–1.90) were considered statistically significant risk factors.

**Table 3:** Multi-predictor logistic regression analysis to evaluate factors associated with CSBD-19 > 50 (n = 5,227).

Variables	β	SE	OR	OR (lower)	OR (upper)	*p*-value
Intercept	-4.56	0.41	0.01	0.00	0.02	**<0.001**
Male	1.20	0.15	3.32	2.45	4.50	**<0.001**
Married/cohabiting	0.03	0.14	1.03	0.79	1.34	0.84
Exhibitionism (sexual interest)	0.54	0.19	1.72	1.18	2.52	**<0.05**
Diaperism/Eproctophilia/Masks/ Scatophilia/Urolagnia (sexual interest)	0.33	0.16	1.39	1.02	1.90	**<0.05**
Zoophilia (sexual interest)	0.87	0.17	2.39	1.70	3.37	**<0.001**
Paraphilia discomfort	0.66	0.15	1.94	1.44	2.61	**<0.001**
Net household income between 1 and 2	0.55	0.17	1.74	1.26	2.40	**<0.001**
Heterosexual	-0.28	0.35	0.75	0.38	1.49	0.42
Homosexual	-0.33	0.37	0.72	0.35	1.47	0.36
Bisexual	0.08	0.34	1.08	0.55	2.11	0.82
18–24 YO	0.07	0.22	1.08	0.69	1.67	0.74
25–30 YO	0.18	0.21	1.20	0.79	1.82	0.40
31–40 YO	0.33	0.19	1.40	0.97	2.01	0.07
Gender Diverse Individuals	-0.40	0.40	0.67	0.30	1.49	0.33
Fetishism (sexual interest)	0.39	0.19	1.47	1.01	2.16	**0.05**
Specific Characteristics (disorder)	0.90	0.25	2.46	1.49	4.04	**<0.001**
Partialism (Disorder)	0.69	0.27	2.00	1.17	3.42	**<0.05**

β = standardized regression coefficients; SE = standard errors; OR = odds ratios; OR (lower)/OR (upper) = 95% confidence intervals of odds ratios. Bold numbers indicate *p*-values considered statistically significant. Sexual Sadism (disorder): B 0.99 / SE 0.21 / OR 2.69 / OR (lower) 1.79 / OR (upper) 4.07 / p-value <0.001. Voyeurism (disorder): B 0.63 / SE 0.23 / OR 1.88 / OR (lower) 1.21 / OR (upper) 2.92 / p-value <0.05.

Regarding protective factors for high risk of CSBD, the model identified three variables with protective tendency, although statistical significance was not maintained after adjusting for all confounders: heterosexuality (OR = 0.75, 95%CI: 0.38–1.49, *p* = 0.42), homosexuality (OR = 0.72, 95%CI: 0.35–1.47, *p* = 0.36) and, gender other than cisgender female or cisgender male (OR = 0.67, 95%CI: 0.30–1.49, *p* = 0.33).

When analyzing the association between the sociodemographic characteristic showed in the **Table 1** (sex at birth, gender identity, age, sexual orientation, marital status, net household income) and high risk of CSBD, several factors demonstrated a trend toward elevated risk; however, after adjustment for potential confounders, these associations were no longer statistically significant. These variables include the following age groups: 31-40 years (OR = 1.40, 95% CI: 0.97–2.01, *p* = 0.07), 25-30 years (OR = 1.20, 95% CI: 0.79–1.82, *p* = 0.40), being at 18-24 years (OR = 1.08, 95% CI: 0.69–1.67, *p* = 0.74), being bisexual (OR = 1.08, 95% CI: 0.55–2.11, *p* = 0.82) and being married or cohabiting (OR = 1.03, 95% CI: 0.79–1.34, *p* = 0.84). The only two variables that reached statistical significance considering those sociodemographic characteristic was being male (OR = 3.32, 95%CI: 2.45–4.50, *p* = <0.001) and net household income between 1 and 2 minimum wages (OR = 1.74 95%CI: 1.26-2.40 (*p* = <0.001).

Lastly, we estimated the positive association between diverse paraphilic interests and disorder and high risk of CSBD. Presenting discomfort regarding any paraphilic interest showed a very strong association (OR = 1.94 95% CI: 1.44-2.61 (p = <0.001), The paraphilic interests that were found to be positively associated were Exhibitionism (OR = 1.72 95% CI: 1.18-2.52 (p = <0.05), Diaperism / Eproctophilia / Masks / Scatophilia / Urolagnia (OR = 1.39 95% CI: 1.02-1.90 (p = <0.05), Zoophilia (OR = 2.39 95% CI: 1.70-3.37 (p = <0.001), Fetishism (OR = 1.47, 95% CI: 1.01–2.16, p = 0.05). Also, two kinds of Paraphilic Disorders showed strong positive association with high risk of CSBD: Body Specific Characteristics (OR = 2.46 95% CI: 1.49-4.04 (p = <0.001) and Partialism Disorder (OR = 2.00 95% CI: 1.17-3.42 (p = <0.05).

## Discussion

The present study aims to contribute to the advancement of research on human sexuality in Brazil by adopting the diagnostic framework established in recent classification systems. To this end, we sought to gather information on the prevalence of CSBD in the Brazilian population studied as it relates to potential risk factors, regarding gender, age, sexual orientation, educational level, acquisitive power, occupation, and the association with paraphilic presentations. We emphasize that this study responds to the call to better establish fundamental parameters to statistically understand the occurrence of CSBD in countries outside North American and European (20, 32).

Our study, which included 5.227 individuals, identified a prevalence of 5.4% (n = 282) for high-risk CSBD and concluded that the CSBD-19 scale retains its psychometric properties: namely, a high level of discrimination and clear differentiation between individuals with and without compulsive sexual behavior. The questions address each specific aspect of the disorder relevant for diagnosis. Therefore, considering the ICD-11 diagnostic criteria, CSBD-19 is a suitable screening tool for CSBs in Brazil and could be used in future studies in our country.

Despite the consistency of these prevalence estimates with prior national (4.35%) and global (4.2%) findings, there remains a limited number of studies examining CSBD through an integrated sociodemographic and clinical framework. For instance, a Brazilian study published in 2013 reported that 72% of patients seeking treatment for compulsive sexual behavior presented with psychiatric comorbidities, particularly anxiety and depression (33, 34). Other studies conducted prior to the introduction of ICD-11 diagnostic criteria have reported similar frequencies (35).

Consistent with prior research, higher rates of compulsive sexual behavior were observed among men (17, 32, 36, 37). A study (38) suggests that gender differences in CSBD may be associated with distinct psychological profiles: women tend to exhibit higher levels of neuroticism and stress vulnerability, whereas men more frequently present comorbid conditions such as depressive disorders, Attention Deficit Hyperactivity Disorder and Obsessive Compulsive Disorder. Cultural factors may also contribute to this disparity. For instance, shame (39) and higher levels of sexual inhibition among women, potentially related to concerns about sexual violence or unintended pregnancy (40), have been proposed as protective factors against compulsive sexual behavior. Nevertheless, the underrepresentation of women in clinical and forensic samples necessitates cautious interpretation of these findings.

When comparing subgroups using logistic regression, we did not find differences regarding sexual orientation and gender diverse individuals. This similarity has been identified in previous studies (17, 41). Elevated scores in these populations may reflect external factors, such as stigma or reduced sexual well-being, rather than intrinsic patterns of compulsive sexual behavior (42). This underscores the importance of careful clinical assessment to avoid potential misclassification.

The relationship between CSBD and age has been understudied, with studies showing both positive and negative associations in specific populations (43, 44) and within certain age groups (45–47). In contrast to the present study, which did not include adolescents, a prior research involving approximately 3,000 participants reported no significant differences in clinical symptoms across age groups, including adolescents, and found that clinical symptoms did not significantly differ among these groups (48). Given the inconsistent findings in the literature and the exclusion of adolescents in the present study, future research should employ longitudinal designs to examine developmental trajectories of CSBD symptoms across the lifespan, particularly during adolescence, a critical period for sexual development.

Although individuals with a household income between one and two minimum wages exhibited elevated risk, this pattern was not observed among those earning below one minimum wage, which may indicate a non-linear relationship between income and CSBD risk. However, it may also reflect sampling bias, given the overrepresentation of individuals with higher socioeconomic status in the sample.

Several limitations should be considered. Using an online convenience sample may have restricted participation to those with internet access and a particular interest in the topic, introducing selection bias. As a result, these findings may not represent the broader Brazilian population. On the other hand, studies conducted using online or convenience samples have enabled the inclusion of certain sociodemographic strata, such as women, whose participation had previously represented a barrier in clinical samples (42, 49). Unmeasured confounding factors, such as psychiatric symptoms and conditions or substance use, could have influenced the observed associations. Additionally, reliance on self-report measures may have introduced reporting bias, particularly given the sensitive nature of investigating sexual behavior. The absence of clinical diagnostic interviews further limits the ability to confirm CSBD diagnoses. The crosssectional design also precludes causal inference.

The study identified a statistically significant association between paraphilic interests and increased risk of CSBD applying bivariate analysis. This association was stronger when paraphilic behaviors approached the threshold of a paraphilic disorder. Previous research has reported co-occurrence of paraphilic disorder and CSBD rates of up to 30% in clinical samples (3, 50). However, the nature of this relationship remains complex and should not be interpreted as indicative of causality. While certain paraphilic interests were associated with higher CSBD risk, others were not, underscoring the heterogeneity of these phenomena. These findings suggest that the relationship between CSBD and paraphilic interests is not uniform, supporting a dimensional rather than categorical interpretation and emphasizing the importance of differentiating between normative atypical interests and clinically significant patterns. Notably, pedophilia, which is among the most significant paraphilias due to its legal implications and risk of recidivism (51, 52), was not found to be associated with a high risk of CSBD in the population studied. According to previous studies, individuals with pedophilic disorder and other paraphilic conditions (such as sexual sadism and exhibitionism) show a considerably higher risk to recidivism when comorbid CSBD is present (53). These observations highlight the need to better understand the heterogeneity of individuals with paraphilic disorders and to examine the complexity of the overlap between CSBD and paraphilic conditions.

The present findings also reinforce the importance of considering socioeconomic and demographic variables when investigating CSBD. Male sex remained independently associated with high-risk CSBD after adjustment for confounding variables, consistent with previous international studies. In contrast, variables such as sexual orientation, gender diversity, marital status, and age lost statistical significance in multivariate analyses, suggesting that some previously observed associations may reflect broader psychosocial or contextual factors rather than independent predictors. The association observed among individuals with lower household income may indicate potential links between socioeconomic vulnerability, psychological distress, reduced access to mental health services, and compulsive sexual behaviors, although this interpretation remains speculative and requires further investigation.

## Conclusion

This study aimed to address previous calls for more inclusive research in the fields of sexuality and compulsive sexual behaviors (10, 11, 21) by including a large Brazilian sample, thereby contributing to the representation of Latin American populations in this field, as well as individuals with diverse sexual orientations and gender identities. In the population we studied, we found that the estimated frequency of high-risk CSB is 5.4%, using the diagnostic framework established in the ICD-11 as a reference. This finding suggests that compulsive sexual behaviors occur at a prevalence comparable to that reported for other mental health conditions, such as anxiety disorders, depressive disorders and substance use disorders (54, 55).

The substantial co-occurrence of PDs and CSBD observed in our sample indicates that when one condition is identified, the co-occurrence observed in this study suggests that clinicians may benefit from assessing both conditions during evaluation. Moreover, our findings indicate that not all paraphilic disorders constitute a risk factor for CSBD, supporting the view that these conditions should be conceptualized separately and that estimates of impairment and potential risk (for example, among sexual offenders) should be evaluated on an individual basis.

The present findings may contribute to future epidemiological and longitudinal investigations examining the developmental trajectories and clinical correlates of CSBD across culturally diverse populations. The identification of specific paraphilic interests and disorders associated with elevated odds of high-risk CSBD may support the development of more targeted screening strategies, dimensional assessment protocols, and preventive interventions in both psychiatric and forensic settings. Furthermore, the psychometric performance of the Brazilian Portuguese version of the CSBD-19 observed in this study supports its potential applicability in future multicenter studies and cross-cultural comparisons involving Latin American populations. Future studies should also investigate the longitudinal stability of these associations, the influence of psychiatric comorbidities and minority stress, as well as possible neurobiological and psychosocial mechanisms underlying the co-occurrence between CSBD and paraphilic disorders.

Although the clinical construct of CSBD has been delineated within the ICD-11 framework, its phenomenological characteristics and its relationship with psychiatric comorbidities remain incompletely understood. Future longitudinal and nationally representative studies are essential to clarify causal pathways and refine diagnostic thresholds for CSBD.

## Data Availability

The raw data supporting the conclusions of this article will be made available by the authors, without undue reservation.
