# Supplementary material for: Compulsive sexual behavior disorder and paraphilic disorders in a large Brazilian community sample: an online investigation
Source: Front Psychiatry. 2026 Oct 7;17:1807692. doi: 10.3389/fpsyt.2026.1807692 (PMC13683565; doi:10.3389/fpsyt.2026.1807692)
Supplement: Supplementary file 1 [file DataSheet1.pdf]

ANEXO C – INSTRUMENTOS DE COLETA DE DADOS

QUESTIONÁRIO SOCIODEMOGRÁFICO

1. **A sua idade corresponde a qual faixa etária?**

   18 – 24 anos

   25 – 30 anos

   31 – 40 anos

   41 – 50 anos

   51 – 60 anos

   Mais de 60 anos

2. **Qual é o seu sexo biológico?**

   Feminino

   Masculino

   Outro [escrever]

3. **Qual é o seu gênero identificado?**

   Feminino

   Masculino

   Outro [escrever]

4. **Qual é a sua orientação sexual?**

   Heterossexual

   Homossexual

   Bissexual

   Outro [escrever]

5. **Qual é, aproximadamente, a renda média mensal do seu núcleo familiar?**
Até 01 salário mínimo

Entre 01 e 02 salários mínimos

Entre 03 e 04 salários mínimos

Entre 05 e 06 salários mínimos

Entre 07 e 08 salários mínimos

Mais de 08 salários mínimos

6. **Qual é o seu estado civil?**
Solteiro (a)

Casado (a) / União Estável

Separado (a) / Divorciado (a)

Viúvo (a)

7. **Qual é a sua profissão?**
[escreva]

8. **Quantos anos de estudo você tem?**
0

1

2

3

4

5

6

7

8

9

10

11

12

13

14

15

16

17

18

19

20

21 ou mais
