# Supplementary material for: Compulsive sexual behavior disorder and paraphilic disorders in a large Brazilian community sample: an online investigation
Source: Front Psychiatry. 2026 Oct 7;17:1807692. doi: 10.3389/fpsyt.2026.1807692 (PMC13683565; doi:10.3389/fpsyt.2026.1807692)
Supplement: Supplementary file 2 [file DataSheet2.pdf]

ANEXO B – TERMO DE CONSENTIMENTO LIVRE ESCLARECIDO

TERMO DE CONSENTIMENTO LIVRE E ESCLARECIDO

CAAE:52810521.0.0000.5327

Você está sendo convidado(a) a participar de uma pesquisa cujo objetivo é estudar alguns comportamentos sexuais na população brasileira e identificar possíveis associações entre variantes de comportamento sexual com potencial prejuízo individual. Esta pesquisa está sendo realizada pelo Serviço de Psiquiatria do Hospital de Clínicas de Porto Alegre (HCPA).

Se você aceitar o convite, sua participação na pesquisa envolverá responder a um questionário on-line sobre dados sociodemográficos e comportamentos sexuais e aspectos da sua infância. O tempo médio de resposta é de 20 minutos.

Os possíveis desconfortos decorrentes da participação na pesquisa são responder a questões que envolvem aspectos da sua intimidade como, por exemplo, fantasias sexuais. Não há garantia de que esta pesquisa, neste momento, traga algum benefício direto para os participantes envolvidos, mas poderá contribuir para o melhor entendimento de características do comportamento sexual da população brasileira adulta. Os resultados desta pesquisa também poderão contribuir para a construção de estratégias de prevenção e redução do prejuízo relacionado a transtornos do comportamento sexual . Sua participação na pesquisa é totalmente voluntária e não está previsto nenhum tipo de pagamento. O questionário será respondido de maneira totalmente anônima. Ou seja, não é necessário identificar-se para respondê-lo.

Caso você tenha dúvidas em relação a esta pesquisa ou a este Termo, a qualquer momento você poderá entrar em contato com a pesquisadora responsável, Dra. Maria Inês Rodrigues Lobato, pelo telefone 51 99711873 ou com a pesquisadora Karine Schwarz, pelo telefone (54) 997109853 ou com Comitê de Ética em Pesquisa

do Hospital de Clínicas de Porto Alegre (HCPA), pelo e-mail cep@hcpa.edu.br, telefone (51) 33596246 ou Av. Protásio Alves, 211 - Portão 4 - 5º andar do Bloco C - Rio Branco – Porto Alegre/RS, de segunda à sexta, das 8h às 17h.

Ao responder que concorda em participar da pesquisa você está será direcionado(a) para o questionário.

Agradecemos a sua participação.
