# Supplementary material for: Compulsive sexual behavior disorder and paraphilic disorders in a large Brazilian community sample: an online investigation
Source: Front Psychiatry. 2026 Oct 7;17:1807692. doi: 10.3389/fpsyt.2026.1807692 (PMC13683565; doi:10.3389/fpsyt.2026.1807692)
Supplement: Supplementary file 3 [file DataSheet3.pdf]

ANEXO A – CONVITE PARA PARTICIPAR DA PESQUISA

Este convite poderá ser enviado por email, whats app, ou estar disponível em redes sociais.

CONVITE PARA PARTICIPAR DE PESQUISA

Você está sendo convidado a participar de uma pesquisa que tem por objetivo investigar alguns comportamentos sexuais na população brasileira e identificar possíveis associações entre variantes de comportamento sexual com potencial prejuízo individual. Se você tem interesse em participar da pesquisa clique aqui [https://docs.google.com/forms/d/e/1FAIpQLSfxTrgrNiNqMnjZkjD-PC8LioGwZa0cyE_-dD7lmqUIxivA9w/viewform] e você será direcionado (a) para o Termo de Consentimento Livre e Esclarecido, documento que contém mais informações sobre a pesquisa. A participação na pesquisa será por meio de resposta a um questionário sobre dados sociodemográficos, comportamentos, fantasias e compulsões sexuais, uso de pornografia e eventos traumáticos da infância.

Se após a leitura do Termo de Consentimento você decidir participar da pesquisa, responda à pergunta: Você concorda em participar da pesquisa?

Ao responder Sim você será direcionado para o questionário. O tempo médio de resposta é de 20 minutos.

Agradecemos o seu tempo e atenção.

Equipe de pesquisa.
