# Supplementary material for: Compulsive sexual behavior disorder and paraphilic disorders in a large Brazilian community sample: an online investigation
Source: Front Psychiatry. 2026 Oct 7;17:1807692. doi: 10.3389/fpsyt.2026.1807692 (PMC13683565; doi:10.3389/fpsyt.2026.1807692)
Supplement: Supplementary file 4 [file DataSheet4.pdf]

ANEXO D – ESCALA CSBD-19

Abaixo existem algumas frases que descrevem diversos pensamentos, sentimentos e comportamentos sobre sexo. Por favor, considere os últimos 6 meses e marque na escala de 1 a 4 o quanto essas afirmações correspondem a você. Não existe resposta correta ou errada. Para o propósito desde questionário, sexo é definido como qualquer atividade ou comportamento que estimula ou excita uma pessoa, com a intenção de produzir um orgasmo ou prazer sexual (como a auto-masturbação, sexo individual, usando pornografia, sexo com parceiro, sexo oral, sexo vaginal, sexo anal, etc). A atividade sexual pode ou não envolver um parceiro.

Considere:

1. **“discordo totalmente”;**
2. **“discordo parcialmente”**
3. **“concordo parcialmente”**
4. **“concordo totalmente”**

1. Apesar do meu comportamento sexual ser irresponsável e inconsequente, eu acho difícil parar.
2. Sexo tem sido a coisa mais importante da minha vida.
3. Eu fui capaz de resistir pouco as minhas necessidades sexuais.
4. Eu fiz sexo mesmo quando eu não estava gostando.
5. Meus impulsos e necessidades sexuais me mudaram de um jeito negativo.
6. Eu não consegui controlar minha fissura e meu desejo sexual.
7. Eu prefiro fazer sexo do que fazer qualquer outra coisa.
8. Tentar reduzir a quantidade de sexo nunca funcionou pra mim.
9. Mesmo que o sexo não me de prazer como antes, eu ainda continuo fazendo.
10. Eu não consigo realizar tarefas importantes por causa do meu comportamento sexual.
11. Meu desejo sexual me controla.
12. Quando eu tenho a oportunidade de fazer sexo, o resto se torna irrelevante.
13. Eu nao consegui reduzir a quantidade de sexo que eu faço.
14. Apesar da minha vida sexual não ser tão satisfatória quanto era antes, eu faco sexo.
15. Minhas atividades sexuais interferem no meu trabalho e educação.
16. Meu comportamento sexual teve um impacto negativo nas minhas relações pessoais.
17. Eu tenho incomodado os outros com meu comportamento sexual.
18. Minhas atividades sexuais prejudicaram na minha capacidade de fazer sexo saudável.
19. Eu frequentemente me coloquei em uma situação constrangedora por causa do meu comportamento sexual.
