# Supplementary material for: Compulsive sexual behavior disorder and paraphilic disorders in a large Brazilian community sample: an online investigation
Source: Front Psychiatry. 2026 Oct 7;17:1807692. doi: 10.3389/fpsyt.2026.1807692 (PMC13683565; doi:10.3389/fpsyt.2026.1807692)
Supplement: Supplementary file 5 [file DataSheet5.pdf]

# ANEXO E - QUESTIONÁRIO DE INTERESSES PARAFÍLICOS

Nesta parte você responderá várias questões íntimas com conteúdo sexual explícito. Você concorda em continuar?

Sim

Não

01. Você se excita sexualmente ao pensar em pessoas com certas características específicas tais como tatuagem, piercing, corpo peludo, corpo extremamente musculoso, obeso, com cicatrizes, machucados, amputações, próteses, deficiências ou outras?

Sim

Não

1.I. Essa fantasia de excitação sexual já dura pelo menos 6 meses?

Sim

Não

1.II. Você se masturba pensando nesta fantasia?

Sim

Não

1.III. Com que frequência você se masturba pensando nesta fantasia?

Raras vezes

Poucas Vezes

Algumas Vezes

Muito Frequentemente

1.IV. Essa fantasia lhe causa algum sofrimento ou incômodo?

Sim

Não

1.V. Esse desejo sexual causa alguma dificuldade na sua vida profissional, social ou afetiva?

Sim

Não

02. Desconsiderando a genitália, você se excita sexualmente pensando em partes específicas do corpo - por exemplo, cabelo, pés, umbigo, barriga, ânus, axila, braços, etc?

Sim

Não

2.I. Essa fantasia de excitação sexual já dura pelo menos 6 meses?

Sim

Não

2.II. Você se masturba pensando nesta fantasia?

Sim

Não

2.III. Com que frequência você se masturba pensando nesta fantasia?

Raras vezes

Poucas Vezes

Algumas Vezes

Muito Frequentemente

2.IV. Essa fantasia lhe causa algum sofrimento ou incômodo?

Sim

Não

2.V. Esse desejo sexual causa alguma dificuldade na sua vida profissional, social ou afetiva?

Sim

Não

03. Você acha alguns objetos ou materiais (por exemplo: sapatos, meias, roupas íntimas, couro, etc.) tão sexualmente excitantes a ponto de precisar deles para se excitar sexualmente?

Sim

Não

3.I. Essa fantasia de excitação sexual já dura pelo menos 6 meses?

Sim

Não

3.II. Você se masturba pensando nesta fantasia?

Sim

Não

3.III. Com que frequência você se masturba pensando nesta fantasia?

Raras vezes

Poucas Vezes

Algumas Vezes

Muito Frequentemente

3.IV. Essa fantasia lhe causa algum sofrimento ou incômodo?

Sim

Não

3.V. Esse desejo sexual causa alguma dificuldade na sua vida profissional, social ou afetiva?

Sim

Não

04. Você já se excitou sexualmente com o pensamento de usar roupas típicas de outro gênero (homens: calcinhas, sutiãs, meias etc. / mulheres: cuecas etc.)?

Sim

Não

4.I. Essa fantasia de excitação sexual já dura pelo menos 6 meses?

Sim

Não

4.II. Você se masturba pensando nesta fantasia?

Sim

Não

4.III. Com que frequência você se masturba pensando nesta fantasia?

Raras vezes

Poucas Vezes

Algumas Vezes

Muito Frequentemente

4.IV. Essa fantasia lhe causa algum sofrimento ou incômodo?

Sim

Não

4.V. Esse desejo sexual causa alguma dificuldade na sua vida profissional, social ou afetiva?

Sim

Não

05. Você já se excitou sexualmente ao pensar em seu/sua parceiro(a) sexual exercendo poder ou exercendo domínio sobre você? Por exemplo, seu/sua parceiro(a) o(a) amarrando, machucando e você se submetendo a ele(a)?

Sim

Não

5.I. Essa fantasia de excitação sexual já dura pelo menos 6 meses?

Sim

Não

5.II. Você se masturba pensando nesta fantasia?

Sim

Não

5.III. Com que frequência você se masturba pensando nesta fantasia?

Raras vezes

Poucas Vezes

Algumas Vezes

Muito Frequentemente

5.IV. Essa fantasia lhe causa algum sofrimento ou incômodo?

Sim

Não

5.V. Esse desejo sexual causa alguma dificuldade na sua vida profissional, social ou afetiva?

Sim

Não

06. Você já se excitou sexualmente com o pensamento de você exercer domínio ou poder sobre alguém? Por exemplo: amarrar, machucar ou humilhar seu/sua parceiro(a)?

Sim

Não

6.I. Essa fantasia de excitação sexual já dura pelo menos 6 meses?

Sim

Não

6.II. Você se masturba pensando nesta fantasia?

Sim

Não

6.III. Com que frequência você se masturba pensando nesta fantasia?

Raras vezes

Poucas Vezes

Algumas Vezes

Muito Frequentemente

6.IV. Essa fantasia lhe causa algum sofrimento ou incômodo?

Sim

Não

6.V. Esse desejo sexual causa alguma dificuldade na sua vida profissional, social ou afetiva?

Sim

Não

07. Você já se excitou sexualmente com a ideia de ver/assistir a outras pessoas em situações íntimas - por exemplo, tomando banho, trocando de roupas ou fazendo sexo - sem que elas saibam que você está olhando?

Sim

Não

7.I. Essa fantasia de excitação sexual já dura pelo menos 6 meses?

Sim

Não

7.II. Você se masturba pensando nesta fantasia?

Sim

Não

7.III. Com que frequência você se masturba pensando nesta fantasia?

Raras vezes

Poucas Vezes

Algumas Vezes

Muito Frequentemente

7.IV. Essa fantasia lhe causa algum sofrimento ou incômodo?

Sim

Não

7.V. Esse desejo sexual causa alguma dificuldade na sua vida profissional, social ou afetiva?

Sim

Não

8. Você já se excitou sexualmente com a ideia de mostrar sua genitália em público para pessoa(s) desconhecida(s) sem o consentimento dela(s) - por exemplo, em frente a um homem, mulher ou crianças?

Sim

Não

8.I. Essa fantasia de excitação sexual já dura pelo menos 6 meses?

Sim

Não

8.II. Você se masturba pensando nesta fantasia?

Sim

Não

8.III. Com que frequência você se masturba pensando nesta fantasia?

Raras vezes

Poucas Vezes

Algumas Vezes

Muito Frequentemente

8.IV. Essa fantasia lhe causa algum sofrimento ou incômodo?

Sim

Não

8.V. Esse desejo sexual causa alguma dificuldade na sua vida profissional, social ou afetiva?

Sim

Não

09). Você já se excitou sexualmente com a ideia de se esfregar em outra(s) pessoa(s) sem o consentimento dela(s) em público ou no meio de uma multidão, por exemplo, em transporte público, em shows, em locais aglomerados etc?

Sim

Não

9.I. Essa fantasia de excitação sexual já dura pelo menos 6 meses?

Sim

Não

9.II. Você se masturba pensando nesta fantasia?

Sim

Não

9.III. Com que frequência você se masturba pensando nesta fantasia?

Raras vezes

Poucas Vezes

Algumas Vezes

Muito Frequentemente

9.IV. Essa fantasia lhe causa algum sofrimento ou incômodo?

Sim

Não

9.V. Esse desejo sexual causa alguma dificuldade na sua vida profissional, social ou afetiva?

Sim

Não

10). Você já se excitou com a ideia de ter práticas sexuais específicas como fazer sexo usando máscaras, usando fraldas, práticas que envolvem peido, urina, fezes ou outras?

Sim

Não

10.I. Essa fantasia de excitação sexual já dura pelo menos 6 meses?

Sim

Não

10.II. Você se masturba pensando nesta fantasia?

Sim

Não

10.III. Com que frequência você se masturba pensando nesta fantasia?

Raras vezes

Poucas Vezes

Algumas Vezes

Muito Frequentemente

10.IV. Essa fantasia lhe causa algum sofrimento ou incômodo?

Sim

Não

10.V. Esse desejo sexual causa alguma dificuldade na sua vida profissional, social ou afetiva?

Sim

Não

11). Você já se excitou sexualmente com o pensamento de fazer sexo com animais?

Sim

Não

11.I. Essa fantasia de excitação sexual já dura pelo menos 6 meses?

Sim

Não

11.II. Você se masturba pensando nesta fantasia?

Sim

Não

11.III. Com que frequência você se masturba pensando nesta fantasia?

Raras vezes

Poucas Vezes

Algumas Vezes

Muito Frequentemente

11.IV. Essa fantasia lhe causa algum sofrimento ou incômodo?

Sim

Não

11.V. Esse desejo sexual causa alguma dificuldade na sua vida profissional, social ou afetiva?

Sim

Não

12). Você já se excitou com a ideia de fazer sexo com cadáveres?

Sim

Não

12.I. Essa fantasia de excitação sexual já dura pelo menos 6 meses?

Sim

Não

12.II. Você se masturba pensando nesta fantasia?

Sim

Não

12.III. Com que frequência você se masturba pensando nesta fantasia?

Raras vezes

Poucas Vezes

Algumas Vezes

Muito Frequentemente

12.IV. Essa fantasia lhe causa algum sofrimento ou incômodo?

Sim

Não

12.V. Esse desejo sexual causa alguma dificuldade na sua vida profissional, social ou afetiva?

Sim

Não

13). Você acha meninos/meninas com corpo infantil ou meninos/meninas com corpo pré-adolescente (sem pelos, sem mamas) sexualmente excitantes?

Sim

Não

13.I. Essa fantasia de excitação sexual já dura pelo menos 6 meses?

Sim

Não

13.II. Você se masturba pensando nesta fantasia?

Sim

Não

13.III. Com que frequência você se masturba pensando nesta fantasia?

Raras vezes

Poucas Vezes

Algumas Vezes

Muito Frequentemente

13.IV. Essa fantasia lhe causa algum sofrimento ou incômodo?

Sim

Não

13.V. Esse desejo sexual causa alguma dificuldade na sua vida profissional, social ou afetiva?

Sim

Não

14). Você acha meninos/meninas no início da adolescência excitantes sexualmente? (meninos com pelos e com saco escrotal em desenvolvimento; meninas com pelos púbicos em desenvolvimento e mamas pequenas)

Sim

Não

14.I. Essa fantasia de excitação sexual já dura pelo menos 6 meses?

Sim

Não

14.II. Você se masturba pensando nesta fantasia?

Sim

Não

14.III. Com que frequência você se masturba pensando nesta fantasia?

Raras vezes

Poucas Vezes

Algumas Vezes

Muito Frequentemente

14.IV. Essa fantasia lhe causa algum sofrimento ou incômodo?

Sim

Não

14.V. Esse desejo sexual causa alguma dificuldade na sua vida profissional, social ou afetiva?

Sim

Não
